# Surgical management, use and efficacy of adjuvant dyes in idiopathic epiretinal membranes: a systemic review with network meta-analysis

**DOI:** 10.1186/s40942-023-00515-3

**Published:** 2023-12-06

**Authors:** Miguel A. Quiroz-Reyes, Erick A. Quiroz-Gonzalez, Miguel A. Quiroz-Gonzalez, Virgilio Lima-Gomez

**Affiliations:** 1 Retina Department of Oftalmologia Integral ABC (Medical and Surgical Nonprofit Organization) Affiliated with the Postgraduate Studies Division at the National Autonomous University of Mexico, Av. Paseo de las Palmas 735 Suite 303, Lomas de Chapultepec, 11000 Mexico City, Mexico; 2 Institute of Ophthalmology, Fundacion Conde de Valenciana (Nonprofit Organization) affiliated with the Postgraduate Studies Division at the National Autonomous University of Mexico, Av. Chimalpopoca 14. Col. Obrera, 06800 Mexico City, Mexico; 3 Juarez Hospital, Public Assistance Institution (Nonprofit Organization), Av. Politecnico Nacional 5160, Colonia Magdalena de las Salinas, 07760 Mexico City, Mexico

**Keywords:** Central macular thickness, Idiopathic epiretinal membranes, Network meta-analysis, Dye-assisted ILM peeling, Dye-stained combined ERM and ILM peeling, Brilliant blue G, Membrane blue-dual

## Abstract

**Background:**

The epiretinal membrane (ERM) is a nonvascular fibrocellular tissue formed by cellular metaplasia and proliferation at the vitreoretinal surface and is generally treated by pars plana vitrectomy (PPV) with or without internal limiting membrane (ILM) peeling. This network meta-analysis aimed to compare the efficacy of all available ERM removal interventions and assessed the use and efficacy of surgical dyes in managing idiopathic ERMs.

**Methods:**

MEDLINE, EMBASE, Cochrane CENTRAL, and the US National Library of Medicine were searched (June 28, 2023). Clinical studies that included patients with ERMs were included. Randomized controlled trials (RCTs) were also appraised using Cochrane risk of bias (ROB).

**Results:**

Ten RCTs and ten non-RCTs were included in this study. A pairwise meta-analysis between ERM removal and combined ERM and ILM removal showed no significant difference in visual outcome (change in BCVA) 1 year postintervention (MD = − 0.0034, SE = 0.16, *p* = 0.832). Similarly, there was no significant difference in the central macular thickness postoperatively between the two groups (MD = − 4.95, SE = 11.11, *p* = 0.656) (Q = 4.85, *df* = 3, *p* = 0.182, I^2^ = 41.21%). The difference in ERM recurrence between the groups was also not statistically significant (OR = 4.64, *p* = 0.062, I^2^ = 0). In a network meta-analysis, there was no significant difference in visual outcomes between ERM removal only and other treatment modalities: combined ILM and ERM removal (MD = 0.039, *p* = 0.837) or watchful waiting (MD = 0.020, *p* = 0.550). In a network meta-analysis, there was no significant difference in the visual outcomes between ERM removal alone and dye-stained combined ERM and ILM peeling (MD = 0.122, *p* = 0.742 for brilliant blue G; BBG and MD = 0.00, *p* = 1.00 for membrane blue-dual; MBD). The probability of being a better surgical dye for better visual outcomes was 0.539 for the MBD group and 0.396 for the BBG group. The recurrence of ERM was not significantly different when the ILM was stained with any of the dyes. No study was judged on ROB assessment as having low ROB in all seven domains.

**Conclusion:**

The two types of surgical modalities provided comparable efficacy, with no significant differences between the outcomes. Among the dye-assisted ILM peeling methods, the membrane blue-dual dye was the most effective in providing better structural and functional outcomes.

**Supplementary Information:**

The online version contains supplementary material available at 10.1186/s40942-023-00515-3.

## Background

The epiretinal membrane (ERM) proliferation is a nonvascular fibrocellular tissue formed by cellular metaplasia and proliferation at the vitreoretinal surface [1]. It causes symptomatic visual disturbances due to retinal wrinkling and distortion [2]. ERM may occur without antecedent ocular conditions or surgical procedures and is termed idiopathic or primary ERM. It may also be associated with retinal vascular diseases, trauma, or surgery and is termed secondary ERM. ERM secondary to retinal vascular diseases is very common and has been reported to be strongly associated with diabetic retinopathy [1]. The pooled age-standardized prevalence estimates of early ERM, advanced ERM, and any ERM have been reported to be 6.5%, 2.6%, and 9.1%, respectively [3]. There have been reports of ethnicity-specific prevalence rates for ERMs, with Chinese ethnicity reported to be at higher risk of contracting the disease [1]. Reported risk factors associated with the development of primary ERMs include old age, longer axial length, smoking, ethnicity, and cataracts [1].

ERM is generally treated by pars plana vitrectomy (PPV) with or without internal limiting membrane (ILM) peeling [2]. Removal of the ILM during ERM surgery has been reported to be useful in preventing subsequent recurrences [4]. However, this maneuver can cause structural and functional macular damage or endanger the macula with iatrogenic complications (tears, bleeding, and retinal pigment epithelium (RPE) damage due to microscope coaxial light or adjuvant dye toxicity). Therefore, some clinicians prefer not to remove the ILM to avoid these complications.

The ERM, ILM, and vitreous humor are semitransparent structures that are difficult to visualize without using vital dyes. Therefore, dyes are commonly used in vitreoretinal surgeries [5]. Different dyes have been used in ERM surgeries to stain the ERM, ILM, or both. However, the efficacy of these dyes in improving the structural and functional outcomes of surgery has not yet been clearly established.

Few systematic reviews have compared different surgical techniques in ERM management; however, these reviews have pooled both randomized and nonrandomized studies [6–8]; therefore, they have been subjected to bias or used a conventional pairwise meta-analysis yielding only one pooled effect estimate [9, 10]. Therefore, the present network meta-analysis (NMA) was conducted to compare the efficacy of all available ERM removal interventions and to assess the use and efficacy of surgical dyes in the management of idiopathic ERM.

## Methods

### Search strategy

The present systematic review was conducted according to the Preferred Reporting Items for Systematic Reviews and Meta-analysis (PRISMA) extension for Network Meta-analysis (PRISMA-NMA) guidelines [11]. A comprehensive search strategy (Additional file 1) was developed to identify articles that reported interventions for the surgical management of ERM. The review protocol was developed before the literature search and strictly followed. The MEDLINE, Embase, Scopus, and Cochrane Library databases were systematically searched from their inception to July 28, 2023, and the search results were limited to English-language articles.

### Study eligibility criteria

The inclusion criteria are listed in Table 1. This review included studies published in English that used surgical procedures to manage idiopathic ERMs in patients aged ≥ 18 years. Meta-analyses were performed for randomized controlled trials (RCTs), in which individuals were randomly assigned to the two treatment groups. Nonrandomized trials (non-RCTs), defined as interventional studies that included investigator-controlled treatment allocation of participants, and case studies, defined as interventional studies that reported descriptive data of individuals, are summarized in tabulated form. Given the potential for bias in case studies and the fact that more rigorous study designs offering stronger evidence were included in this review, the case studies were not synthesized. Conference abstracts, retrospective studies, and reviews were also excluded.

**Table 1:** PICO criteria for inclusion of studies

Population	Clinical cases, ≥ 18 years of age, with diagnosed idiopathic ERM who were surgically managed
Intervention	Surgical intervention (ERM removal) without ILM peeling
Comparator	Surgical intervention (ERM removal) with ILM peeling and/or any other interventions for ERM
Outcomes	Outcome measures
	Postoperative BCVA in logMAR at 6–12 months
	Recurrence rate at 1 year
	Efficacy of different transoperative adjuvants; for example, BBG, TB, ICG/IFCG, MBD

BBG, Brilliant Blue G; BCVA, best-corrected visual acuity; ERM, epiretinal membrane; ILM, internal limiting membrane; ICG, indocyanine green; IFCG, infracyanine green; logMAR, logarithm of the minimum angle of resolution; MBD membrane blue-dual; PICO, population, intervention, comparator, outcomes; TB, trypan blue

### Selection of studies and data extraction

Citations retrieved from different databases were imported into the Covidence [12], a systematic review tool used for screening, selection, and data extraction. Two reviewers (MAQR and EAQG) independently screened the articles based on their titles and abstracts, followed by a full-text review. Any discrepancy in the inclusion of articles was resolved through consensus, or a third reviewer (MAQG) was consulted when consensus could not be reached.

Two independent review authors (MAQR and VLG) extracted the data. The extracted data were recorded using a specially designed data-extraction form. The extracted data included the study’s first author and year of publication, study location, study design, mean age, standard deviation (SD) of the participants in each group, sample size, follow-up period (months), type of intervention(s), adjuvant dyes used, and outcome measures.

### Data analysis

Data analysis was performed using R software version 4.3.1, which consists of a traditional pairwise meta-analysis using the *meta* [13] and *metafor* [14] packages, whereas a network meta-analysis (NMA) was performed using the *netmeta* package [15]. The results are presented using forest plots. The interventions were ranked using P scores, and the corresponding surface under the cumulative ranking curve scores (SUCRA) values were also recorded [16]. Summary estimates for continuous outcomes were reported as the mean difference (MD), while categorical outcomes were reported using the odds ratio (OR). Statistical significance was set at *p* < 0.05.

### Assessment of heterogeneity

A random effects model was used to conduct the NMA, and heterogeneity was assessed using the Cochrane Chi-square and I^2^ statistics. An I^2^ value greater than 50% indicated substantial heterogeneity, as described in the Cochrane Handbook of Systematic Reviews [17]. In addition, the design inconsistency in the network was assessed using the decomposition of Cochrane’s Q statistic into between (inconsistency) and within design (heterogeneity) variability in the effect sizes using the *netmeta* package.

### Assessment of risk of bias

Risk of bias (ROB) assessment was independently performed for the RCTs by two authors (MAQR and VLG). Disagreements were resolved through consensus. The Cochrane ROB tool [18] was used to appraise RCTs (Additional file 2). The Cochrane ROB tool comprises seven domains; each domain was judged as having i) low, ii) unclear, or iii) high ROB.

## Results

**Fig. 1:**
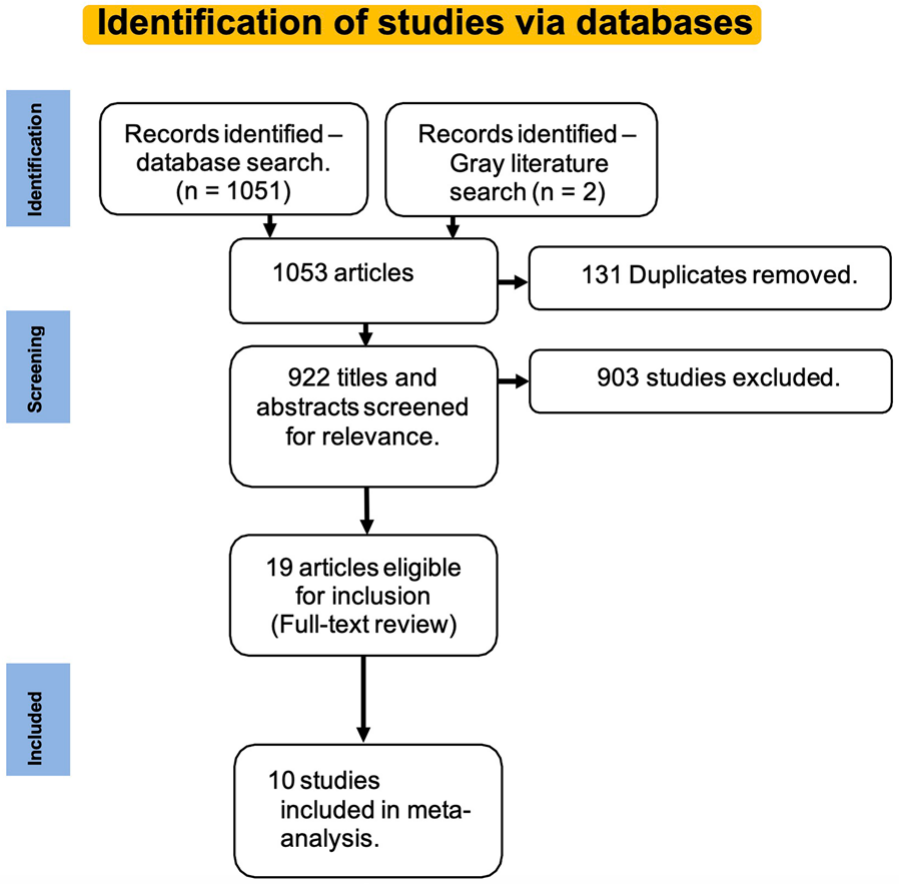
PRISMA flow chart outlining the article selection process

The literature search yielded 1053 articles (146 from MEDLINE, 16 from Embase, 889 from Scopus, 1 from the Cochrane Library, and 1 from the gray literature search), of which 19 were eligible for inclusion. A PRISMA flowchart is presented in Fig. 1. Ten RCTs [19–28], one prospective comparative nonrandomized trial [29] and eight prospective interventional studies [30–37] were included in this qualitative synthesis. However, only RCTs were included in this meta-analysis.

In the included prospective studies, the interventions mainly included PPV with ERM peeling only, with or without the use of a surgical dye, or combined ERM and ILM peeling with an adjuvant surgical dye. In the included RCTs and nonrandomized comparative studies, ERM peeling was compared to combined ERM and ILM peeling. One RCT [20] compared ERM removal with watchful waiting for ERM for a year. A range of surgical dyes have been used in these studies, including indocyanine green (ICG), infracyanine green (IFCG), trypan blue (TB 0.06 or 0.15%), brilliant blue G (BBG), triamcinolone acetonide (TA), view ILM, and combination dyes, such as membrane blue-dual (MBD).

Different outcome measures were assessed for the included studies. The outcome measures were best-corrected visual acuity at different follow-up periods, central macular thickness (CMT), metamorphopsia, and ERM recurrence. The characteristics of the included RCTs are presented in Table 2A, and those of the non-RCTs are presented in Table 2B.

**Table 2:** Characteristics of included (a) RCTs, (b) non-RCTs

References	Study place	Mean age (years) ± SD group1/group2	Sample size	Follow-up (months)	Type of intervention	Adjuvants dye(s)	Outcome measures
(A)							
Hillenkamp et al*.* [19]	Germany	67.0 ± 7.0	60	4	PPV with ERM peeling without ICG versus with ICG	ICG	BCVA, metamorphopsia, CMT, and residual or recurrent macular ERM
		71.0 ± 6.0					
Ripandelli et al*.* [28]	Italy	72.3 ± 8.3	60	12	PPV with ERM peeling versus ERM + ILM peeling	BBG for ILM peeling	Central retinal sensitivity, BCVA and OCT parameters
Kofod et al*.* [20]	Denmark	69.0 ± 3.0	53	12	PPV with ERM peeling versus Watchful waiting	BBG for ERM peeling group	BCVA, and CMT
		66.0 ± 6.0					
Sola et al*.* [21]	Korea	70.6 (53.0–81.0)	22	24	PPV with ERM peeling versus ERM + ILM peeling	TB in all patients	ILM extraction pattern, BCVA, CMT, ERM recurrence, and adverse events
		(Median values)					
Tranos et al*.* [22]	Greece	70.0 ± 6.0	102	12	PPV with ERM peeling versus ERM + ILM peeling	TB 0.15% for ERM and BBG for ILM peeling	Mean change in BCVA (distance and near), change in metamorphopsia and change in SD-OCT characteristics
		68.0 ± 12.0					
DeNovelli et al*.* [23]	Brazil	66.0 ± 9.6	63	6	PPV with ERM peeling versus ERM + ILM peeling	BBG for ILM only	Functional and anatomical outcome
		67.0 ± 9.4					
Russo et al*.* [24]	Italy	72.7 ± 7.5	38	12	PPV with ERM peeling versus ERM + ILM peeling	Combination of 0.025% BBG and 0.15% TB (MBD) both groups	Foveal and perifoveal retinal sensitivity, visual acuity, and CMT, and adverse ocular events
		69.8 ± 6.5					
Aydin et al*.* [25]	Turkey	67.5 ± 5.9	36	4	PPV with ERM peeling versus ERM + ILM peeling	TB 0.06% both groups	Metamorphopsia, BCVA and macular volume
		67.7 ± 6.2					
Jatoi et al*.* [26]	Pakistan	NA	44	12	PPV with ERM peeling versus ERM + ILM peeling	NA	BCVA and CMT
Gabriel et al*.* [27]	Austria	69.0 ± 8.0	51	3	PPV with ERM peeling versus ERM + ILM peeling	(MBD) for both groups	Mean SCP, FAZ, CMT, retinal volume, and BCVA
		70.0 ± 5.0					
(B)							
Arndt et al*.* [30]	France	63.4 ± 9.2	104	12	PPV with ERM peeling	NA	Metamorphopsia, BCVA
Garweg et al*.* [29]	Switzerland	70.1 ± 5.2	43	12	PPV with ERM + ILM peeling	IFCG 0.5% for group 1 and 0.15% TB for group 2	BCVA (distance and near), macular visual-field indices
		70.3 ± 7.5					
Kinoshita et al*.* [31]	Japan	70.0 ± 0.9	75	24	PPV with ERM + ILM peeling	TA	Metamorphopsia, BCVA, and OCT parameters
Shahzadi et al*.* [32]	Pakistan	72.0 ± 5.0	30	6	PPV with ERM removal without ILM peeling	NA	BCVA, CMT and foveal thickness on SD OCT, and recurrence of ERM
Ehlers et al*.* [33]	United States	68.4	76	12	PPV with ERM peeling + optional ILM peeling	ICG and/or TA in all participants	BCVA, CST, and complications including ERM recurrence
Scupola et al*.* [34]	Italy	72 ± 14.5	49	12	PPV with ERM + ILM peeling	TA for ERM and BBG Peel for ILM	SANFL, and long term decrease of RNFL thickness
Jonna et al*.* [35]	United States	67.4 ± 5.6	40	60	PPV with ERM peeling	TA and/or ICG	RNFL layer and GC-IPL thicknesses using SD-OCT
Zobor et al*.* [36]	Austria	71.1 ± 6.3	54	3	PPV with ERM + ILM peeling	View-ILM dye	Choroidal thickness, CRT, BCVA
Datlinger et al*.* [37]	Austria	71.0 ± 6.7	32	3	PPV with combined ERM and ILM peeling	View-ILM (Alchimia, Italy)	Changes in PFD before (baseline) and after surgery

(a) BBG, Brilliant Blue G; BCVA, best-corrected visual acuity; CMT, central macular thickness; FAZ, foveal avascular zone; ERM, epiretinal membrane; ICG, indocyanine green; ILM, internal limiting membrane; MBD, membrane blue-dual; NA, not available; PPV, pars plana vitrectomy; SD, standard deviation; SD-OCT, spectral domain-optical coherence topography; RCTs, randomized controlled trials; SCP, superficial capillary plexus; TB. Trypan blue

(b) BBG, Brilliant Blue G; BCVA, best-corrected visual acuity; CRT, central retinal thickness; CST, central subfield thickness; ERM, epiretinal membrane; GC-IPL, ganglion cell-inner plexiform layer; ICG, indocyanine green; IFCG, infracyanine green; ILM, internal limiting membrane; NA, not available; OCT, optical coherence topography; PPV, pars plana vitrectomy; PFD, papillo-foveal distance; SD-OCT, spectral domain-optical coherence tomography; RCTs, randomized controlled trials; RNFL, retinal nerve fiber layer; SANFL, swelling of the arcuate nerve fiber layer; SD, standard deviation; TA, triamcinolone acetonide; TB, trypan blue

### Efficacy of interventions

#### Comparison of different modalities of ERM peeling

In the included studies, there were three types of interventions for the surgical management of ERM: PPV with ERM removal, PPV with ERM along with ILM removal, and watchful waiting of ERM for 1 year.

There were four RCTs [20, 22–24] from which complete data could be extracted to assess the visual outcome between 6 months and 1 year postintervention. A pairwise meta-analysis between ERM removal and combined ERM and ILM removal showed no significant difference in visual outcome (change in BCVA) 1 year postintervention (MD = − 0.0034, SE = 0.16, *p* = 0.832). There was no significant heterogeneity among studies (Q = 3.82, *df* = 3, *p* = 0.281, I^2^ = 0.07%). Similarly, there was no significant difference in the central macular thickness postoperatively between the two groups (MD = − 4.95, SE = 11.11, *p* = 0.656) (Q = 4.85, *df* = 3, *p* = 0.182, I^2^ = 41.21%). The difference in ERM recurrence between the groups was not statistically significant (OR = 4.64, *p* = 0.062, I^2^ = 0).

**Fig. 2:**
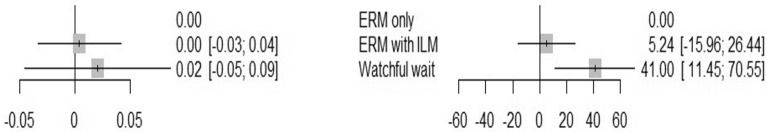
Changes in BCVA and central macular thickness in the ERM with ILM and watchful wait groups (Reference group ERM peeling only group; MP)

In a network meta-analysis, there was no significant difference in visual outcomes between ERM removal only and other modalities of treatment, namely, combined ILM and ERM removal (MD = 0.039, *p* = 0.837) or watchful waiting (MD = 0.020, *p* = 0.550). No significant heterogeneity (I^2^ = 22.2%) was observed in the network. The probability of each intervention being the best was 0.653, 0.540, and 0.307 for ERM alone, ERM with ILM, and 0.307 for watchful waiting, respectively. Based on 1000 simulations, the SUCRA values were 0.645, 0.544, and 0.310, respectively. Similarly, CMT was not significantly different between the ERM-only and ERM-with-ILM groups (MD = 5.24, *p* = 0.628), whereas a significant difference was observed in the watchful waiting group (MD = 41.00, *p* < 0.006, I^2^ = 38.2%). The probabilities of each intervention being the best were 0.841, 0.643, and 0.015 for the ERM-only, ERM-with-ILM, and watchful waiting groups, respectively. The corresponding SUCRA values are 0.837, 0.654, and 0.009, respectively. Forest plots for the ranking of each intervention (ERM with ILM and watchful waiting) compared with ERM peeling only (reference group) for visual outcome and changes in CMT are presented in Fig. 2.

#### Network meta-analysis for the most appropriate surgical dye for ERM removal

In the RCTs included in the analysis, ILM was stained with BBG, ICG, and MBD dyes. In a network meta-analysis, there was no significant difference in the visual outcomes between ERM removal alone and dye-stained ERM combined with ILM peeling (MD = 0.122, *p* = 0.742 for BBG and MD = 0.00, *p* = 1.00 for MBD). The probability of being a better surgical dye for better visual outcomes were 0.539 and 0.396 in the MBD and BBG groups, respectively. The corresponding SUCRA values were 0.528 and 0.406, respectively. Similarly, there was no significant difference in CMT when the ILM was stained with BBG (MD = 5.49, *p* = 0.705) or MBD (MD = − 2.00, *p* = 0.939, I^2^ = 54.1%). The probability score for MBD was 0.564, whereas for BBG, it was 0.377.

**Fig. 3:**
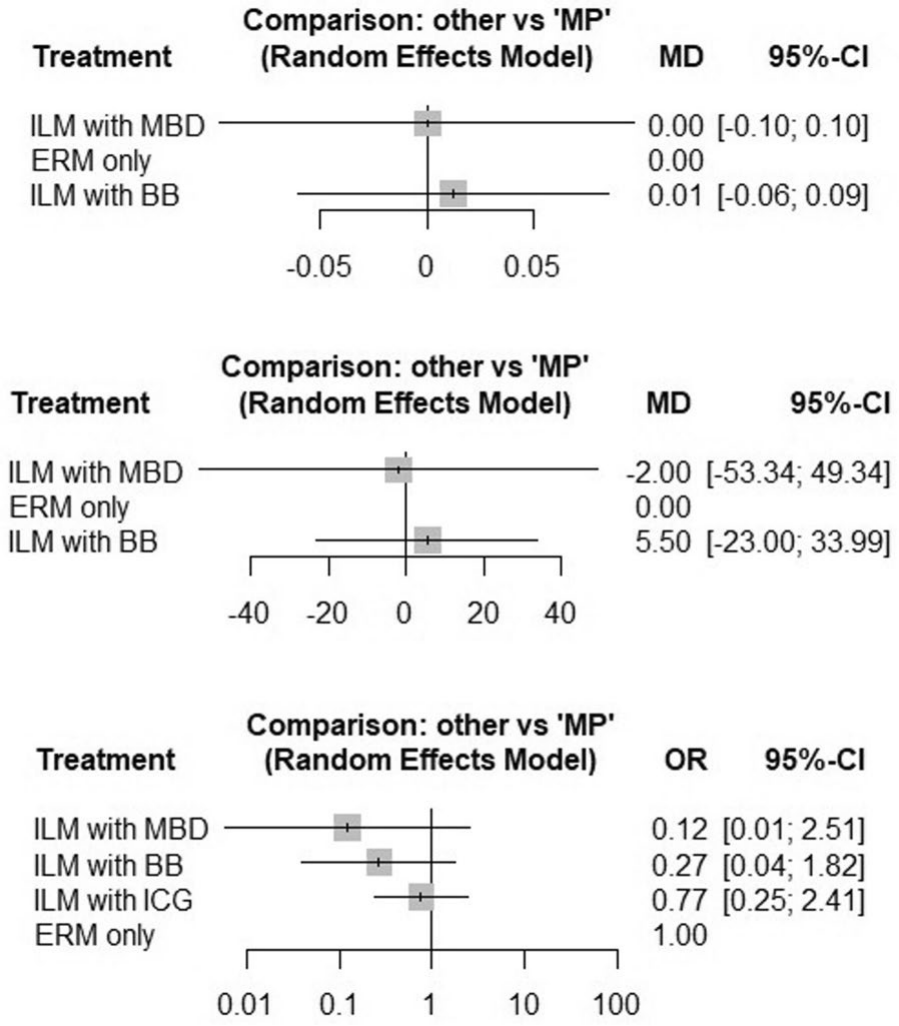
Changes in BCVA, central macular thickness and ERM recurrence with different dyes (BBG, ICG and MBD) (Reference group ERM peeling; MP)

In a network meta-analysis, the recurrence of ERM was not significantly different when the ILM was stained with any of the dyes, namely, MBD (OR = 0.12, *p* = 0.172), BBG (OR = 0.27, *p* = 0.179), or ICG (OR = 0.77, *p* = 0.653). There was no evidence of heterogeneity (I^2^ = 0) in the network. The probabilities of being the best dye for nonrecurrence of ERMs were 0.817, 0.687, and 0.327 for MBD, BBG, and ICG, respectively. The corresponding SUCRA values based on 1000 simulations were 0.806, 0.689, and 0.345, respectively. Forest plots showing the effects of different dyes on the visual outcome, change in CMT, and recurrence of ERMs are presented in Fig. 3.

#### Complications

Intraoperative or postoperative complications related to ERM with or without ILM surgery were reported in six studies: two RCTs, one nonrandomized comparative study, and three prospective studies. The reported complications are summarized in Table 3. Retinal breaks and/or retinal detachment (RD), cystoid macular edema (CME), ocular hypertension, and cataract progression are commonly reported complications. We were unable to perform a meta-analysis due to insufficient data.

**Table 3:** Complications related to ERM removal in the included studies

References	Study design	Type of surgery	Adjuvant dye(s)	Complications
Ehlers et al*.* [34]	Prospective interventional	PPV with ERM peeling with optional ILM peeling at surgeon's discretion	ICG and/or TA	Cataract progression (3/52 phakic patients) RD (1/76)
Hillenkamp et al*.* [19]	RCT	PPV with ERM peeling with versus without ICG	ICG	RD (2/27 in ICG; 1/32 in without ICG group) paracentral, lower nasal visual field defect (2/27 in ICG group)
Jonna et al*.* [36]	Prospective interventional	PPV with ERM peeling	ICG and/or TA at surgeon's discretion	PVD (5/20) ocular hypertension (1/20)
Shahzadi et al*.* [33]	Prospective interventional	PPV with ERM peeling	None	RD (1/30) CME (1/30)
Sola et al*.* [21]	RCT	PPV with ERM peeling only versus ERM with ILM peeling	TB for ERM and BBG for ILM	Intraoperative retinal breaks (1/26) CME with subsequent ocular hypertension (2/26) nonarteritic anterior ischemic optic neuropathy (1/26)
Mackenzie et al*.* [30]	Prospective nonrandomized comparative	PPV with ERM peeling with stain versus without stain	TB 0.15%	Mild retinal hemorrhage 3/16 in stained and 8/18 nonstained participants retinal breaks 1/18 nonstained participants

BBG, Brilliant Blue G; CME, cystoid macular edema; ERM, epiretinal membrane; ICG, indocyanine green; PPV, par plana vitrectomy; PVD, posterior vitreous detachment; RCT, randomized controlled trial; RD, retinal detachment; TA, triamcinolone acetonide; TB, trypan blue

#### Risk of bias assessment

**Fig. 4:**
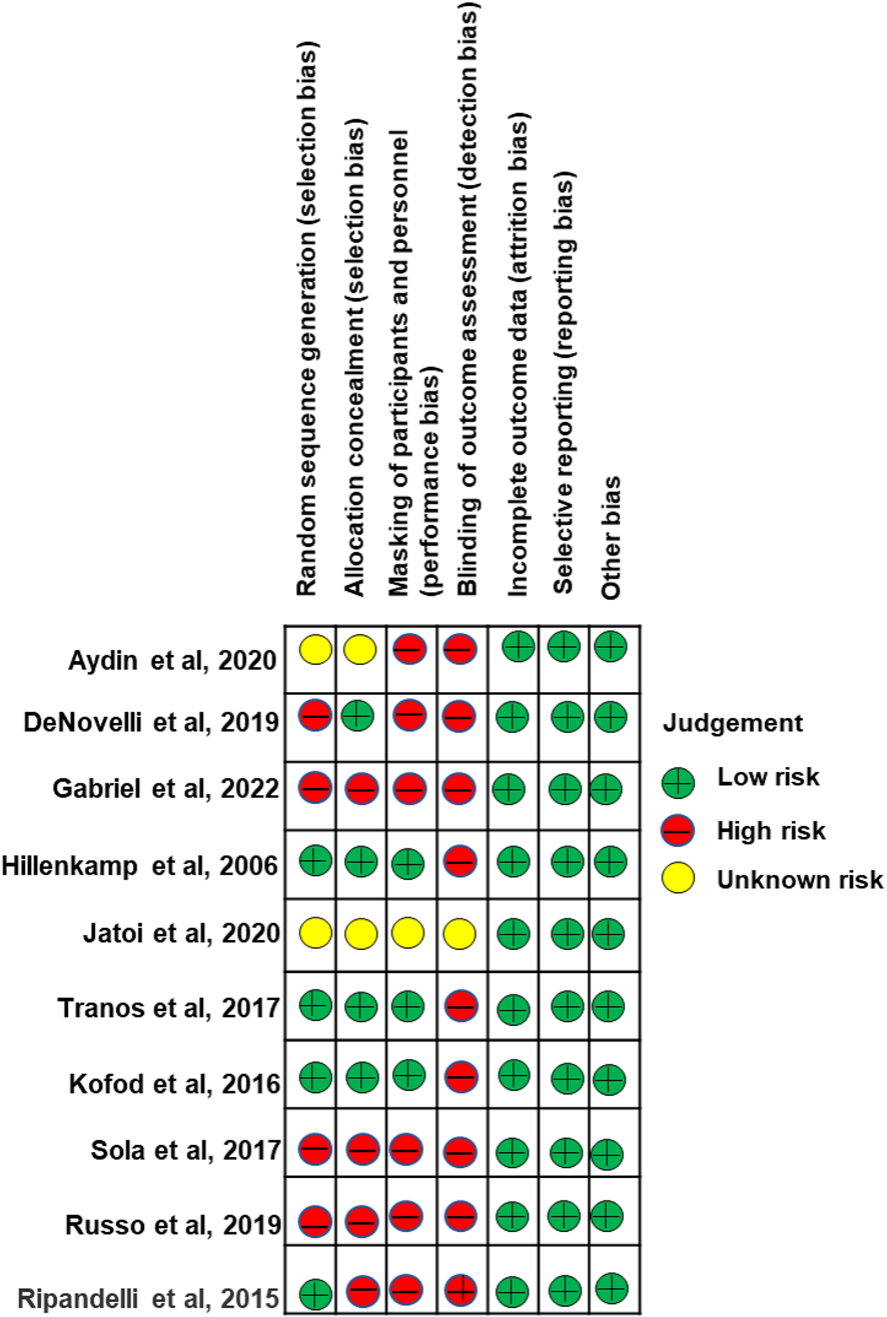
Risk of bias assessment of included RCTs. Green indicates a low ROB, red indicates a high ROB, and yellow indicates an unknown ROB

Figure 4 summarizes the ROB assessments of the RCTs. No study was judged to have low ROB in any of the seven domains. The domains with a lower risk of bias across all studies were ROB due to other sources of bias (10/10 studies), blinding of outcome assessors (10/10 studies), and attrition bias (10/10 studies). The domain with the highest risk of bias was ROB because of masking of participants and personnel (9/10 studies). A summary of the RCTs’ ROB judgments is provided in Additional file 2.

## Discussion

The present systematic review and network meta-analysis aimed to assess the different surgical modalities of treatment and the use and efficacy of adjuvant dyes for idiopathic ERMs. The review identified three major treatment modalities: PPV with ERM removal alone, PPV with combined ERM and ILM removal, and watchful waiting for ERM. Similarly, a range of surgical dyes have been used in previous studies. The dyes used to stain ERM or ILM included TB at concentrations of 0.06% or 0.15%, ICG/IFCG, ILM view, BBG, and MBD dyes.

The NMA showed that both structural (evidenced by changes in CMT and/or recurrence of ERM) and functional (evidenced by visual outcomes) outcomes were not significantly different when the ERM was removed in isolation or when it was removed along with the ILM. However, the CMT increased significantly when no intervention (watchful waiting) was introduced. Although ERM recurrence was lower in the combined ERM and ILM peeling group, the difference was not statistically significant. The ranking of different treatment modalities suggests that although ERM alone and ERM in combination with ILM produced comparable outcomes, watchful waiting for ERM was the least effective modality of treatment for idiopathic ERM. These results of visual outcome and CMT changes in our NMA are in accordance with the pairwise meta-analyses conducted by Far et al*.* [9], Sun et al*.* [10] and an umbrella review conducted by Zhang et al*.* [38] However, unlike these three reviews, we did not find a significant difference in the recurrence of ERM between the groups. Previous meta-analyses had significant limitations because they pooled data from both randomized and nonrandomized studies, including retrospective studies [6–8].

The efficacy of the surgical dyes used for staining ILM did not produce statistically significant differences in the outcomes. However, the MBD dye produced better visual outcomes and lower ERM recurrences rates than the BBG and ICG dyes. Although limited by the number of trials included in the review, this NMA provides an important indication that MBD is a potentially more effective dye for the surgical management of ERM. This finding is concurrent with the published literature about the potential efficacy of MBD in the intraoperative identification of ERMs and ILMs from the surrounding intraocular structures [39]. Similarly, in another recent study, an improved identification of ILM at retinal breaks with MBD was reported [40]. Our findings and concurrent literature indicate a potential role of MBD in managing ERM surgery. Further studies comparing the different dyes are required to confirm these results.

All ten RCTs examining the treatment outcomes of ERM surgeries were identified as having a high risk of performance and detection biases. Surgical intervention in retinal diseases is likely to vary across patients owing to the nature of individual conditions, causes of the disease and many other factors beyond the control of a surgeon. Therefore, masking participants and personnel is difficult because clinicians and patients are typically involved in clinical decision making. Although there was a high risk of bias across the four domains (sequence generation, selection process, masking, and blinding of outcome measures), three other domains (attrition, reporting, and other biases) were judged to be at a low risk. Therefore, the validity of the current NMA results can be useful in making judgments during ERM management surgical planning.

The strength of our NMA is that we included only RCTs in the quantitative synthesis. We assessed the structural and functional outcomes only in idiopathic ERMs. ERMs due to other causes were purposefully excluded so that participants with similar inclusion criteria were included in the NMA, which helped to satisfy the assumption of transitivity. However, this study has several limitations. The number of RCTs included in the review was small, with varying outcome measures. We could not assess the heterogeneity on some occasions because of the limited number of studies.

## Conclusion

A systematic search of the literature identified three treatment modalities for ERM: PPV with ERM removal alone, PPV with ERM and ILM removal, and watchful waiting for ERM. The two types of surgical modalities provided comparable efficacies, with no significant differences between the outcomes. Among the dye-assisted ILM peeling techniques, the MBD dye was the most effective in providing better structural and functional outcomes. Further multicentric RCTs comparing multiple dyes are required to confirm these findings.

### Supplementary Information

**Additional file 1:** Search strategy.

**Additional file 2:** ERM_ROB analysis.

## Data Availability

The datasets used in this study have been included in the main text. Photographs and figures from this study may be released via a written application to the Photographic Laboratory and Clinical Archives Retina Department at the Oftalmologia Integral ABC Medical and Surgical Assistance Institution (Nonprofit Organization) Av. Paseo de las Palmas 735 suite 303, Lomas de Chapultepec, Mexico City 11000, Mexico, and the corresponding author upon request. The search strategy for the data analysis and ERM-ROB analysis can be found in Additional files 1 and 2, respectively.

## Abbreviations

BBG: Brilliant blue G
BCVA: Best-corrected visual acuity
CME: Cystoid macular edema
CMT: Central macular thickness
CST: Central subfield thickness
CRT: Central retinal thickness
ERM: Epiretinal membrane
FAZ: Foveal avascular zone
GC-IPL: Ganglion cell-inner plexiform layer
ICG: Indocyanine green
IFCG: Infracyanine green
ILM: Internal limiting membrane
logMAR: Logarithm of the minimum angle of resolution
MBD: Membrane blue-dual
MD: Mean deviation
MP: Most-powerful test
NMA: Network meta-analysis
OCT: Optical coherence tomography
OR: Odds ratio
PFD: Papillo-foveal distance
PICO: Population, intervention, comparator, outcomes
PPV: Pars plana vitrectomy
PRISMA: Preferred reporting items for systematic reviews and meta-analysis
PRISMA-NMA: Preferred reporting items for systematic reviews and meta-analysis extensions for network meta-analysis
PVD: Posterior vitreous detachment
RCT: Randomized controlled trials
RD: Retinal detachment
RNFL: Retinal nerve fiber layer
ROB: Risk of bias
RPE: Retinal pigment epithelium
SANFL: Swelling of the arcuate nerve fiber layer
SCP: Superficial capillary plexus
SE: Standard error
SD: Standard deviation
SD-OCT: Spectral domain-optical coherence tomography
SUCRA: Surface under the cumulative ranking curse scores
TA: Triamcinolone acetonide
TB: Trypan blue
