# Supplementary material for: Surgical management, use and efficacy of adjuvant dyes in idiopathic epiretinal membranes: a systemic review with network meta-analysis
Source: Int J Retina Vitreous. 2023 Dec 6;9:77. doi: 10.1186/s40942-023-00515-3 (PMC10702105; doi:10.1186/s40942-023-00515-3)
Supplement: Supplementary file 1 — Additional file 1: Search strategy. [file 40942_2023_515_MOESM1_ESM.docx]

**Supplementary information 1**

**Search strategy**

Search query:

( ALL ( epiretinal AND membrane AND surgery OR peeling OR non AND peeling ) AND

ALL ( clinical AND trials ) ) AND ( LIMIT-TO ( DOCTYPE , &quot;ar&quot; ) ) AND ( LIMIT-TO (

LANGUAGE , &quot;English&quot; ) ) AND ( LIMIT-TO ( EXACTKEYWORD , &quot;Human&quot; ) OR

LIMIT-TO ( EXACTKEYWORD , &quot;Epiretinal Membrane&quot; ) OR LIMIT-TO (

EXACTKEYWORD , &quot;Clinical Article&quot; ) OR LIMIT-TO ( EXACTKEYWORD ,

&quot;Controlled Study&quot; ) OR LIMIT-TO ( EXACTKEYWORD , &quot;Internal Limiting

Membrane&quot; ) OR LIMIT-TO ( EXACTKEYWORD , &quot;Internal Limiting Membrane

Peeling&quot; ) OR LIMIT-TO ( EXACTKEYWORD , &quot;Surgical Technique&quot; ) OR LIMIT-TO (

EXACTKEYWORD , &quot;Prospective Study&quot; ) OR LIMIT-TO ( EXACTKEYWORD ,

&quot;Clinical Trial&quot; ) OR LIMIT-TO ( EXACTKEYWORD , &quot;Randomized Controlled Trial&quot; )

OR LIMIT-TO ( EXACTKEYWORD , &quot;Epiretinal Membrane Peeling&quot; ) OR LIMIT-TO (

EXACTKEYWORD , &quot;Article&quot; ) OR LIMIT-TO ( EXACTKEYWORD , &quot;Prospective

Studies&quot; ) OR LIMIT-TO ( EXACTKEYWORD , &quot;Outcome Assessment&quot; ) OR LIMIT-TO

( EXACTKEYWORD , &quot;Postoperative Complications&quot; ) )
